# Association between Childhood Trauma, Mental Health Symptoms and Adherence Among Youth Living with HIV in Botswana

**DOI:** 10.1007/s40653-024-00658-x

**Published:** 2024-08-29

**Authors:** Keneilwe Molebatsi, Vuyokazi Ntlantsana, Merrian J. Brooks, Esther Seloilwe

**Affiliations:** 1 Department of Psychiatry, Faculty of Medicine, University of Botswana, Gaborone, Botswana; 2 Department of Psychiatry, Nelson Mandela School of Medicine, Faculty of Health Sciences, University of Kwazulu Natal, Durban, South Africa; 3 Department of Pediatrics, University of Pennsylvania Perelman School of Medicine, Philadelphia, Pennsylvania USA; 4 Craig Dalsimer Division of Adolescent Medicine, Children’s Hospital of Philadelphia, Philadelphia, Pennsylvania USA; 5 UPENN Partnership, Gaborone, Botswana; 6 School of Nursing, Faculty of Health Sciences, University of Botswana, Gaborone, Botswana

**Keywords:** Trauma, Adherence, HIV, Mental health

## Abstract

**Background:**

People living with HIV experience traumatic incidents at higher rates than the general population; and research has documented significant association between trauma exposure and the development of mental disorders. Mental health problems have a a negative impact on anti-retroviral treatment adherence. All of these psychosocial concerns play a role in potentially increasing HIV transmission to sexual partners resulting in increased incidence rates. To inform interventions that improve quality of life, and decrease risky behaviors for adolescents and youth, it is critical to understand the extent to which childhood trauma, mental health problems, and poor adherence occur and coexist in this population of adolescents living with HIV. Thus, this study examined the associations between childhood trauma, mental health problems (depression, anxiety, and substance use) and adherence to ART among HIV infected youth in Botswana.

**Methods:**

A cross-sectional quantitative survey was conducted among youth aged 15 and 24-years old living with HIV. The Childhood Trauma Questionnaire- Short Form; Depression, Anxiety, Stress Scale (DASS-21; Car Relax Alone Forget Friends Trouble” or CRAFFT 2.1 + N were used to collect data on exposure to childhood trauma, mental health symptoms and problematic substance use respectively. Adherence was assessed objectively with viral load and subjectively with Simplified Medication Adherence Questionnaire. Data analysis was conducted using Stata version 15. Bivariate logistical regression analysis testing for associations between mental health symptoms, substance use and adherence, and childhood trauma was conducted. Multivariate logistic regression was subsequently performed controlling for variables found to be significantly associated with childhood trauma.

**Results:**

Of the 119 youth, 47% of the participants reported experiencing at least one type of childhood trauma, and physical neglect was the most frequently reported. Emotional abuse, emotional neglect, physical abuse, sexual abuse and physical neglect were significantly associated with non-adherence, OR 5.83; OR 3.10; OR 5.97, and OR 2.52, respectively.

**Conclusion:**

Our findings revealed that exposure to all domains of childhood trauma except physical abuse were significantly associated with non-adherence. Sexual abuse and emotional neglect had the highest odds of predicting non-adherence. This highlights the need for trauma focused psychosocial interventions in managing youth living with HIV.

**Supplementary Information:**

The online version contains supplementary material available at 10.1007/s40653-024-00658-x.

## Introduction

Young people aged 10–24 years emerge as the most vulnerable population to the Human Immunodeficiency Virus/Acquired Immunodeficiency Syndrome (HIV/AIDS) (Olashore et al., 2022); with 2.1 million adolescents (10–19 years) living with HIV in Sub- Saharan Africa (UNICEF, 2019a). Although the use of Antiretroviral Therapy (ART) to treat HIV has significantly reduced AIDS-related morbidity and mortality, success is closely associated with consistent and correct use of ART medication.

Despite having the fourth highest HIV prevalence globally (UNAIDS, 2017), Botswana has had a successful fight against HIV/AIDS (UNAIDS, 2018). However, incidence has reportedly gone up among the age group of 15–24 years (UNICEF, 2019b). The high rates of new infections may be explained by the significantly low levels of HIV knowledge among young people, with under half (47%) of those between 15 and 24 able to answer basic questions on HIV (UNAIDS, 2016). Other causes might include vulnerability to sexual assault, STI’s that increase likelihood of converting to HIV once exposed, and high-risk behaviours such as inconsistent condom use, multiple concurrent partners and substance use with sexual activity (Mabaso et al., 2021).

The few studies that have been conducted in Botswana highlight the challenges faced by adolescents living with HIV (ALWHIV). Mullan et al. (2015) identified behavior problems, family issues, and HIV medication adherence; Lowenthal et al. (2012), found that psychosocial dysfunction in older children and adolescents was common and was associated with virologic failure. Older adolescents were found to be more likely to have poor adherence with challenges to adherence including concerns about stigma and discrimination, medication side effects and denial of HIV status (Lowenthal et al., 2012; Mullan et al., 2015a; Yang et al., 2018).

Persons living with HIV (PLWHIV) are exposed to high levels of trauma in childhood and adulthood, often at rates greater than those experienced by the general population (Brezing et al., 2015; Closson et al., 2016). Traumatic experiences include abuse, neglect, parental separation, and familial violence. Adverse conditions during childhood may interfere with children’s fundamental physical, emotional, and social development and place them at risk for psychological problems (Cortina et al., 2012). Evidence has established an association between adversities during childhood and the development of mental disorders, with most research coming from the developed world (McLaughlin et al., 2012; Scott et al., 2011). Childhood adversities range from various forms of abuse (physical, sexual, psychological, and neglect), parental loss in terms of death, divorce, or separation, parental mental illness or substance use, and poverty (Committee on Child Maltreatment Research, Policy, and Practice for the Next Decade: Phase II; Board on Children, Youth, 2014; Oladeji et al., 2010).

Research has documented substantially higher prevalence of childhood sexual and physical abuse, exposure to other traumatic experiences and subsequent post-traumatic stress disorder (PTSD) among both men and women living with HIV/AIDS compared with nationally representative general population samples (LeGrand et al., 2015; Whetten et al., 2008). A systematic review by Brezing et al. (2015) confirmed high rates of trauma in people living with HIV infection with rates ranging from 10 to 90%. The review also found that trauma was associated with increased HIV-risk behaviour and poor adherence to ART, these factors contribute to transmission and acquisition of the virus and negative HIV-related outcomes.

Traumatic stressors have been linked to poor health related quality of life (Nightingale et al., 2011) for example, exposure to trauma was found to predict disengagement from care among adolescents living with HIV in Kenya (Enane et al., 2021). Disengagement from care is likely to result in poor adherence with consequent poor health outcomes. The sequelae of exposure to trauma; Post traumatic stress disorder (PTSD) has also been reported to be highly prevalent among PLWHIV who have been exposed to trauma compared to the general population (Kimerling et al., 1999). Patients with PTSD are at an increased risk of substance use disorders (SUD) (Jacobsen et al., 2001) and the co-occurrence of the disorders further contributes to poor health outcomes through several pathways such as poor adherence to ART (Brief et al., 2010) and immune dysregulation (Julnes et al., 2016; Leserman, 2008).

Literature in sub-Saharan Africa found that people living with HIV reported a greater number of childhood and lifetime traumatic experiences than the community cohort and had worse current mental health and health-related physical functioning (Pence et al., 2012). In Botswana, Seloilwe and Thupayagale-Tshweneagae (2009) found that exposure to sexual abuse and violence by adolescent girls had negative mental health consequences, such as depression and anxiety. (Seloilwe & Thupayagale-Tshweneagae, 2009).

The high rates of trauma exposure in PLWHIV predict elevated rates of co-occurring mental health problems in PLWHIV compared to the general population (Kamau et al., 2012; Ramaiya et al., 2016; Remien et al., 2019; Waldron et al., 2021; Wang et al., 2018). Depression, anxiety and substance use are experienced at disproportionately high rates among PLWHIV (Remien et al., 2019). Mental health problems increase risk of poor treatment adherence and participation in risky behaviours (Chandwani et al., 2011; Dow et al., 2016; Kim et al., 2014; Uthman et al., 2014). Adherence to ART is critical to maintaining viral suppression and preventing morbidity and mortality among HIV-infected patients. Compared to younger children and adults, HIV-positive adolescents demonstrate consistently higher rates of poor adherence to ART and virologic failure (Dow et al., 2014; Kahana et al., 2013; Lowenthal et al., 2014; Mellins & Malee, 2013; Mullan et al., 2015b; Nachega et al., 2009). A systematic review by MacPherson et al. (2015) found that only 62% of 12 to 24-year-olds achieved 95% or greater adherence, anything less than 95% adherence has been associated with negative health outcomes among HIV positive patients (Iacob et al., 2017).

Whetten et al., (2013) found that poor ART adherence was more likely to be reported amongst participants who experienced a greater number of childhood traumatic events such as sexual abuse prior to puberty and the death of an immediate family member. Those with poor adherence had higher depressive symptom severity and post-traumatic stress disorder.

Mental health problems have been found to have a negative impact on ART adherence among HIV positive adolescents (Vreeman et al., 2017). HIV infected adolescents who do not adhere to treatment, were found to have high viral load with significant association between non-adherence and substance use, as well as internalizing behaviour problems (Chandwani et al., 2011). A systematic review by Vreeman et al. (2017) found that among HIV-infected children and adolescents, depression and anxiety symptoms have been associated with lower adherence to ART.

In addition to their effects on adherence, trauma, mental health and substance use problems place this population at high risk for poor health and quality of life outcomes. All of these psychosocial concerns play a role in potentially increasing HIV transmission to sexual partners resulting in increased incidence rates (Mellins & Malee, 2013). In order to inform interventions that improve quality of life, and decrease risky behaviours for adolescents and youth, it is critical to understand the extent to which childhood trauma, mental health problems, and poor adherence occur and coexist in this population of adolescents living with HIV. Thus, this study examined the associations between childhood trauma, mental health problems (depression, anxiety, and substance use) and adherence to ART among HIV infected youth in Botswana.

## Methodology

### Study Design and Population

A cross-sectional quantitative survey of youth aged 15 and 24-years old living with HIV was conducted. The WHO definition of youth was adopted for this study (World Health Organisation, 2020). All the young people receiving HIV services from the study centre and able to comprehend the study tools in English or Setswana were recruited into the study. We excluded those who had an acute or unstable physical illness at the time of screening.

### Study Site

The study was conducted at a public district hospital located 38 km from Gaborone the capital city of Botswana. The hospital provides physical, psychological, and spiritual services. The study was conducted specifically at the HIV clinic which is run by nursing staff and medical officers. The youth were consulted on only one day of the week at the clinic, therefore data was collected only on that day.

### Sample Size Determination

Sample size was calculated using Epi info version 7 at 95% CI, 50% expected prevalence as suggested in literature when there is no known prevalence (Daniel & Cross, 2018) and a finite population of 170 youths receiving services from the study site at the time of data collection. This yielded a minimum sample size of 119.

### Measures

In this study we assessed self-reported trauma, frequency of substance use, symptoms of common mental disorders, and ART adherence.

#### Socio-Demographics

The researchers designed a questionnaire to capture demographic and clinical information such as age, gender, level of education, duration of antiretroviral treatment.

#### Childhood Trauma

We assessed childhood trauma using the Childhood Trauma Questionnaire- Short Form (CTQ-SF) a 28 question self-report measure of five domains of childhood trauma including emotional abuse, physical abuse, sexual abuse, emotional neglect and physical neglect (Bernstein et al., 2003). For example, on the sexual abuse scale, respondents are asked to respond to statements such as “when growing up, someone tried to make me do sexual things or watch sexual things”. Answers are scored on a 5-point Likert scale from 1-5, with one being never true to five being very often true. For each domain, a score of 0-14 and 15 and above is interpreted as absence and presence of the abuse type, respectively. The instrument has demonstrated good psychometric properties among diverse populations including in Africa (Essien et al., 2018; Gerdner & Allgulander, 2009; Jewkes et al., 2010; Kim et al., 2013; Spies et al., 2019). .

#### Substance Use Disorders

We assessed substance use using the “Car Relax Alone Forget Friends Trouble” or CRAFFT 2.1 + N self-administered tool. The CRAFFT was created to identify substance use disorders in youth aged 12 to 21 years old, including abuse of alcohol, marijuana, and other drugs (Knight et al., 1999). The first part of the tool (Part A) asks about the number of days of alcohol, marijuana, and other drug use in the past 12 months. Part B has six “yes/no” questions in each of the CRAFFT domains. For example, asking if one has ridden in a car driven by someone who was high due to alcohol or drugs, or if substances have been used to feel better and a “yes” answer to two or more questions is scored as problematic substance use needing further assessment. For Part B, a maximum score of six yes choices is possible. The CRAFFT has demonstrated acceptable sensitivity and specificity for identifying adolescents with substance-related problems (Knight et al., 2002). The CRAFFT has not been used in Botswana previously however, it has been shown to be valid across diverse cultural settings(Knight et al., 2003) including in Africa (Gamarel et al., 2017).

#### Common Mental Disorders

We assessed common mental disorders namely, depression and anxiety using the Depression, Anxiety, Stress Scale (DASS-21). The DASS is a 21-item self-report instrument designed to assess negative emotional states of depression, anxiety, and stress (Lovibond & Lovibond, 1995). Respondents respond to questions such as “I couldn’t experience positive feelings at all”, “I felt I was close to panic”, “I found it hard to wind down”, on a 4-point Likert scale from 0 to 3, with zero being never and three being almost always. Depression, anxiety, and stress are then assessed in five categories: normal, mild, moderate, severe and extremely severe. For example, depression scores of 0–9 are categorized as normal ;10–13 as mild; 14–20 and 21–42 as moderate and severe respectively. For our analysis DASS scores were further dichotomized to negative and positive. Any scores categorized as normal or mild were classified as negative for mental health symptoms and scores categorized as moderate to extremely severe were classified as positive for mental health symptoms. The DASS has demonstrated good discriminant validity, acceptable internal consistency, and concurrent validity (Antony et al., 1998) including among youth (Le et al., 2017; Szabó, 2010) and populations of similar cultural identities to Botswana (Dreyer et al., 2019; Moya et al., 2022). We used the brief 21 item version of the DASS instead of the standard 42 item version in the current study because of its brevity whilst maintaining the same factor structure and giving similar results to the full DASS (Antony et al., 1998).

### ART Adherence

Adherence to ARTs was assessed objectively and subjectively. We collected the most recent viral load (collected between one and six months ago) as an objective proxy for adherence. Values < 400 copies/mL and ≥ 400 copies/L were categorised as suppressed and unsuppressed, respectively. Our subjective assessment of adherence was the Simplified Medication Adherence Questionnaire (SMAQ): The SMAQ comprises six questions which assess intentional non-adherence, unintentional non-adherence and frequency of non-adherence. The questions are: (1) Do you ever forget to take your medicine? (2) Are you careless at times about taking your medicine? (3) Sometimes if you feel worse, do you stop taking your medicines? (4) Thinking about the last week, how often have you not taken your medicine? (5) Did you not take any of your medicine over the past weekend? and (6) Over the past three months, how many days have you not taken any medicine at all? Adherence was scored as a “no” response to questions 1, 2, 3 and 5, zero response for question 4 and any response less than 2 for question 6. The sensitivity of the SMAQ in detecting non-adherence was found to be 72% (95% CI 58–86) among a large cohort of HIV infected patients in Spain (Knobel et al., 2002). The SMAQ demonstrated sufficient reliability and validity in HIV-positive Ethiopian women of reproductive age who are on ART(Agala et al., 2019) and has been used to assess adherence among YLWHIV in South Africa (Hirasen et al., 2020).

### Data Collection Procedures

#### Translation and Adaptation of data Collection Instruments

The CTQ-SF, CRAFFT 2.1 + N and the SMAQ were translated to Setswana by a bilingual mental health expert. A linguistic expert was then invited to back translate the tools to English. Discrepancies between the original and the back translated instruments were solved by a consensus between the translator, mental health researchers and clinicians to ensure conceptual equivalence and cultural appropriateness. The final version was piloted among a population (*n* = 10) similar to the target population to verify understanding and clarity of the questionnaire.

### Recruitment and Consenting Procedures

Participants were recruited consecutively by a trained research assistant when they came for clinical review (including refills, and regular clinical assessments) until we reached the sample size. Those who volunteered to participate in the study were directed to a research assistant in a private room. Informed consent was obtained before commencing with data collection for those 18years and above. For participants below the age of 18, consent was requested from their parents, and they assented.

### Data Analysis

After data collection, data entry and quantitative statistical analysis was conducted using Stata version 15. Descriptive analysis of the data was conducted using frequencies and percentages and measures of central tendencies. Prevalence rates of childhood trauma, depression, anxiety, and substance use were presented using proportions. The results of childhood trauma were stratified by gender. Bivariate logistical regression analysis testing for associations between mental health symptoms, substance use and adherence, and childhood trauma was conducted. Multivariate logistic regression was subsequently performed controlling for variables found to be significantly associated with childhood trauma. Participants were dichotomized into two groups for each of the five categories of abuse and neglect. For each category of abuse, participants with scores in the none-mild range were classified as negative for exposure and those with scores in the moderate-severe range were classified as positive for exposure. Participants were further divided into two groups, those who were scored positive for exposure for one or more childhood trauma domains were classified into one group, and those who scored negative for all domains grouped together.

Similar to the scoring classification for childhood trauma domains, mental health symptoms scores in the moderate- extremely severe groups were classified as a positive screen.

## Results

### Participant Characteristics

A total of 119 adolescents were recruited. Table 1 presents the sociodemographic and clinical characteristics of the entire cohort, along with the differences between those with no childhood trauma experiences and those with one or more experiences. The sample was predominantly female (59.66%), most of the participants were in secondary school, 51.26% were adherent to their ART and 87.39% were virologically suppressed.

**Table 1:** Participants demographic and clinical characteristics

Variable		Total cohort	No CTQ	≥ 1 CTQ	*p*-value	Test
N		119	62	57		
Gender*	female	71 (61.21%)	38 (62.30%)	33 (60.00%)	0.80	Pearson’s chi-squared
	male	45 (38.79%)	23 (37.70%)	22 (40.00%)		
Age in years, median (IQR)		20.00 (18.00, 23.00)	20.00 (18.00, 23.00)	21.00 (19.00, 23.00)	0.60	Wilcoxon rank-sum
Education*	primary	4 (3.45%)	2 (3.33%)	2 (3.57%)	0.74	Pearson’s chi-squared
	secondary	73 (62.93%)	39 (65.00%)	34 (60.71%)		
	college/uni	35 (30.17%)	18 (30.00%)	17 (30.36%)		
	other	4 (3.45%)	1 (1.67%)	3 (5.36%)		
ART duration in years, median (IQR)		11.00 (4.00, 15.00)	12.00 (6.00, 15.00)	9.50 (3.00, 15.00)	0.50	Wilcoxon rank-sum
Current VL*	< 400	104 (92.86%)	55 (93.22%)	49 (92.45%)	0.87	Pearson’s chi-squared
	> 400	8 (7.14%)	4 (6.78%)	4 (7.55%)		
Adherence*	adherent	61 (51.69%)	41 (66.13%)	20 (35.71%)	< 0.001	Pearson’s chi-squared
	non-adherent	57 (48.31%)	21 (33.87%)	36 (64.29%)		
Depression	neg	78 (65.55%)	49 (79.03%)	29 (50.88%)	0.001	Pearson’s chi-squared
	pos	41 (34.45%)	13 (20.97%)	28 (49.12%)		
Anxiety	neg	70 (58.82%)	43 (69.35%)	27 (47.37%)	0.015	Pearson’s chi-squared
	pos	49 (41.18%)	19 (30.65%)	30 (52.63%)		
Stress	neg	101 (84.87%)	56 (90.32%)	45 (78.95%)	0.084	Pearson’s chi-squared
	pos	18 (15.13%)	6 (9.68%)	12 (21.05%)		

^*variables have missing values^

### Mental Health Symptoms and Problematic Substance use

Data for the prevalence of mental health symptoms are presented in Table 1. Forty-one (34.45%) participants met criteria for depression, 49 (41.18%) and 18 (15.13%) participants met criteria for anxiety and stress respectively. Problematic substance use was detected in 28 (23.53%) of the participants.

### Internal Consistency of Measures

Internal consistency (Table 2) was generally within acceptable limits except for the physical neglect domain of childhood trauma (38%) and for subjective assessment of adherence (39%).

**Table 2:** Internal consistency of data collection instruments

Tool	Domain	Cronbach’s alpha ()
CTQ	Emotional abuse	0.69
	Physical abuse	0.75
	Sexual abuse	0.91
	Emotional neglect	0.79
	Physical neglect	0.38
DASS	Depression	0.85
	Anxiety	0.79
	Stress	0.79

### Experiences of Childhood Trauma

**Fig. 1:**
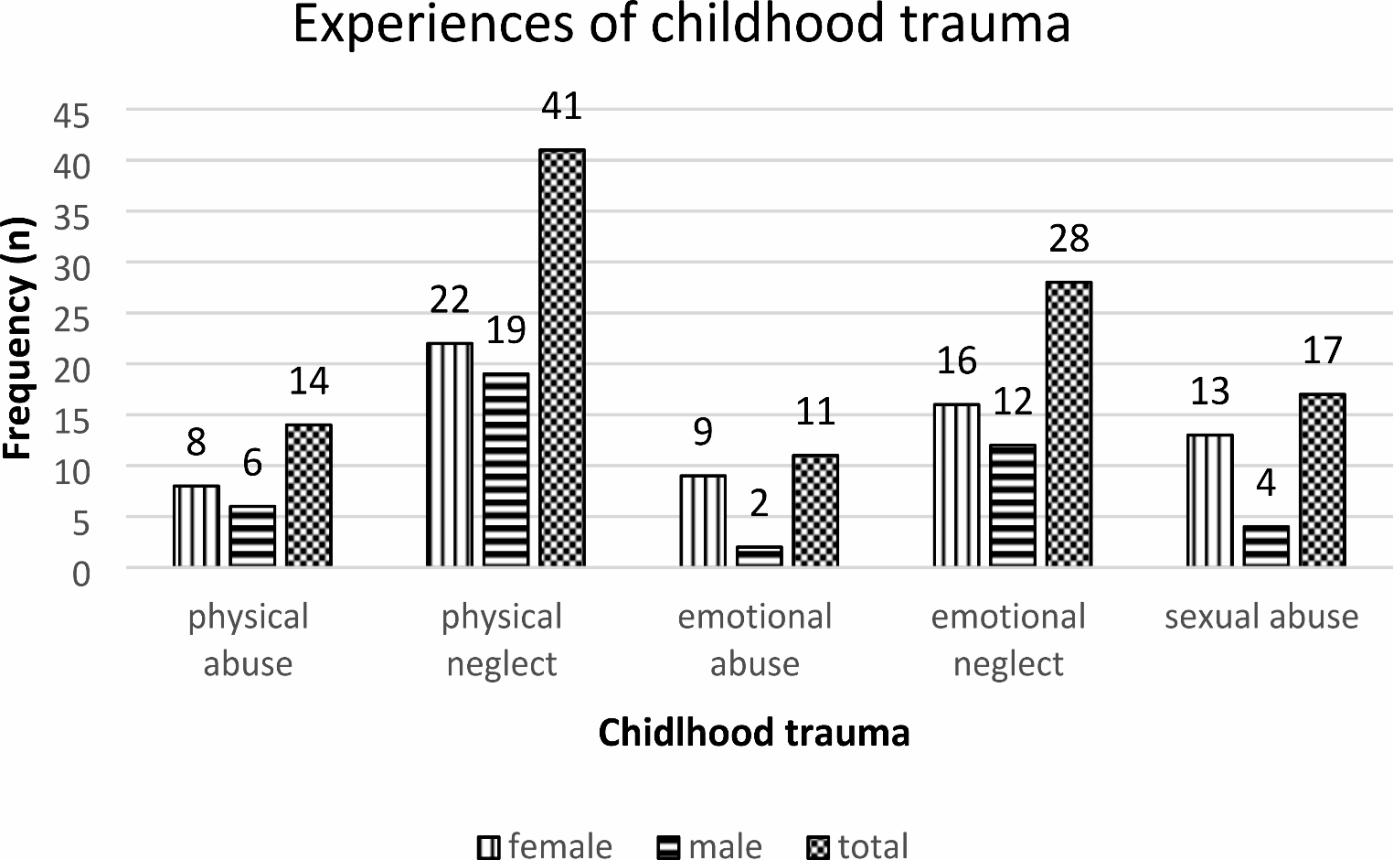
Prevalence and pattern of childhood trauma

Of the 119 youth, 56 (47%) reported experiencing at least one type of childhood trauma. The number of youths reporting each type of trauma by gender is presented in Fig. 1, with multiple types sometimes reported by the same youth. Physical neglect was the most frequently reported childhood trauma subtype with 41 (34.4%) of the 119-youth reporting it. Nineteen (15.97%) of the participants had been exposed to one type of childhood trauma while 26 (21.85%) had been exposed to two types of childhood trauma and 12 (10.80%) had been exposed to three or more types. That is, of the population reporting trauma, 38 (31.93%) had experienced two or more categories of trauma.

### Factors Associated with Childhood Trauma

The bivariate regression model of the association between each category of childhood trauma as the independent variable and mental health symptoms, ART non-adherence, and problematic substance use as dependent/outcome variables were conducted. For each of the outcome variables found to be significantly associated with various categories of childhood trauma, multivariate regression models, controlling for age and gender were created. The results are presented in Table 3. Supplemental Table 1A presents the results of the regression model for each outcome variable. Emotional abuse, emotional neglect, physical abuse, sexual abuse and physical neglect were significantly associated with non-adherence, OR 5.83, *p* = 0.03; OR 3.10, *p* = 0.02; OR 5.97, *p* = 0.008 and OR 2.52, *p* = 0.022 respectively. Mediation analysis of mental health outcomes in the context of childhood trauma are presented in Table 4. There was no evidence of mediation by mental health outcomes.

**Table 3:** Multivariate regression models of association clinical features and childhood trauma, controlling for age and gender

Clinical variable	CTQ	OR	SE	*p*	95%	CI
Depression	≥ 1 Childhood trauma exposure	4.02	1.74	0.001	1.72	9.41
	Emotional abuse	4.33	3.02	0.036	1.10	16.96
	Physical abuse	7.74	5.17	0.002	2.09	28.65
	Physical neglect	3.01	1.27	0.009	1.31	6.90
Anxiety	≥ 1 Childhood trauma exposure	2.65	1.04	0.013	1.23	5.73
	Emotional abuse	4.15	2.97	0.047	1.02	16.92
	Physical abuse	7.58	5.31	0.004	1.92	29.91
Stress	Emotional abuse	8.25	5.16	0.001	2.42	28.10
Substance use	Sexual abuse	8.25	5.16	0.001	2.42	28.10
Non-adherence	≥ 1 Childhood trauma exposure	3.26	1.27	0.003	1.51	7.01
	Emotional abuse	5.83	4.75	0.03	1.18	28.82
	Emotional neglect	3.10	1.45	0.016	1.24	7.73
	Physical abuse	3.14	1.96	0.068	0.92	10.69
	Sexual abuse	5.97	4.04	0.008	1.59	22.48
	Physical neglect	2.52	1.01	0.022	1.15	5.53

**Table 4:** Total indirect effect/Mediation effect of mental health on childhood trauma to outcomes

Variables	Estimate	SE	Est.SE	*p*-value
Childhood trauma - depression- non-adherence	0.061	0.060	1.010	0.312
Childhood trauma - anxiety- non-adherence	0.020	0.042	0.475	0.634
Childhood trauma - stress- non-adherence	0.028	0.046	0.609	0.542
Childhood trauma - depression-substance use	0.028	0.055	0.504	0.614
Childhood trauma - anxiety-substance use	0.059	0.117	0.504	0.614
Childhood trauma - stress-substance use	0.028	0.055	0.504	0.614

## Discussion

Our findings revealed that exposure to all domains of childhood trauma except physical abuse were significantly associated with adherence. Sexual abuse and emotional neglect had the highest odds of predicting non-adherence.

Similar to other studies in Africa (Jewkes et al., 2010; Peltzer, 2007; Suliman et al., 2009; Tucker et al., 2019); this current study found high rates of childhood trauma among YLWHIV. All domains of childhood trauma were associated with mental health symptoms. This is consistent with findings of a meta-analysis by Humphreys and colleagues who found that all forms of child abuse were associated with higher risk of depression, with emotional abuse and neglect having a stronger association compared to other domains of childhood trauma (Humphreys et al., 2020).

In this study physical and emotional neglect were the most common traumatic experiences reported at 35.34% and 24.14% respectively. Emotional neglect has been reported to be prevalent but often goes unreported (Spinazzola et al., 2014; Young et al., 2011). Although the rates found in our sample are lower, the findings compare to a report by Gibbs and colleagues where emotional neglect was also reported by a majority of the participants (75.2%) (A. Gibbs et al., 2018). Gibbs and colleagues may have reported higher rates because there has been evidence suggesting informal settlements are sites of vulnerability for childhood trauma (Meinck et al., 2015). However, our findings contrast with a study conducted in South Africa where exposure to violent traumatic experiences was the most reported (Closson et al., 2016b). While the trauma assessment tool used in the current study did not specifically assess for community violence, unlike South Africa, Botswana has not experienced any political turmoil and crime rates are lower and this may explain the differences in exposure to violent traumatic experiences between the two countries.

Sexual abuse was reported by 14.66% of the participants. This is lower than rates of 36% in women and 47.9% in men that have been reported in South Africa (M. Gibbs et al., 2015). This variance may be explained by the way the two studies reported trauma exposure. In South Africa they considered any exposure as a positive screen whereas this study considered a positive screen as those who reported moderate to severe exposure. Additionally, in this study females experienced sexual abuse at rates higher than males which is comparable to studies conducted elsewhere (Closson et al., 2016a; Richter et al., 2014; Spies & Seedat, 2014).

Exposure to childhood trauma predicted mental health symptoms. This reflects a global body of evidence (Allen et al., 2014; Closson et al., 2016b; Dale et al., 2015; LeGrand et al., 2015; Spies & Seedat, 2014). Shao et al. (2020) in their cohort of a comparable age also found an increase in age to be associated with depression. This was however, not found in our cohort.

Physical abuse and emotional abuse predicted the experience of depression and anxiety. Childhood trauma is associated with sensitization of the neuroendocrine stress response, glucocorticoid resistance, increased central corticotropin-releasing factor (CRF) activity, immune activation, and reduced hippocampal volume (Heim et al., 2008). These neuroendocrine changes reflect risk to develop depression in those whose neural circuitry fail to compensate in response to the challenge by the stress brought about by childhood trauma (Heim et al., 2008).

Similar to what we found, a study on adolescents in Ethiopia found a significant association between physical neglect and depression with children who had experienced parental physical neglect 2.61 times more likely to experience depression than those who had not experienced it (Tirfeneh & Srahbzu, 2020).

In contrast to the meta-analysis by Infurna et al. where they found a strong association between emotional abuse and neglect, and depression (Infurna et al., 2016); we only found an association between depression and emotional abuse but not emotional neglect. We did however, find an association between emotional neglect and anxiety. In youth, anxiety is known to increase the risk of development of depression (Garber & Weersing, 2010).

Having a substance use problem was positively correlated with sexual abuse. This is similar to findings from the United States (U.S.), where participants reported using substances to cope with abuse-related distress (Clum et al., 2009); and Kenya where substance use disorders were found to be significantly associated with adverse childhood experiences (Kiburi et al., 2018).

### Strengths and Limitations

The following limitations should be considered when interpreting findings of this study. Our study enrolled youths presenting to an HIV clinic, indicating they were seeking care. A community sample that included youth lost to follow up and those who are not would have given the true extent of the problem of childhood trauma among those living with HIV since some of these youth may have also already dropped out of care (Belete et al., 2019). Although the CTQ-SF may be subject to a recall bias, it has been found to be both valid and reliable in other adolescent African populations (Essien et al., 2018). Furthermore, screening positive for psychological symptoms is unlikely to have affected reporting of childhood trauma exposure because retrospective reports of childhood trauma have been found to be reliable, independent of the mental state of the respondent (Pinto et al., 2014). Lastly, the findings on the physical neglect domain of childhood trauma need to be interpreted with caution as the internal consistency of this item was very low. Neglect as a concept can be difficult to elucidate as different contexts may define it in different ways(Lansford et al., 2015) for example wearing dirty clothing, may be interpreted as neglect in high resource settings while in low resource settings this may purely be consequence lack of access to water.

## Conclusion

The study highlights that although ARVs have made inroads in sub-Saharan African populations, promoting adherence and overall well-being must include an emphasis on trauma exposure and mental health. Further studies should explore the longitudinal relationship between these factors in the lives of YLWHIV to see if there are any causal mechanisms that can be explored. Additionally, a study of protective factors would also provide targets to promote in psychosocial support programs for these youth. Further, upstream measures such as society wide engagement are recommended for all youth but particularly for vulnerable populations such as YLWHIV. Society wide engagement might include social protections for families who have members living with HIV. Interventions such as cash transfers, parenting classes, community activities for children and youth, and mentoring programs have all shown promise in preventing child trauma. As we can see in this work, there is much to gain from protecting youth from trauma and treating their overall mental health.

## Electronic Supplementary Material

Below is the link to the electronic supplementary material.

Supplementary Material 1

## Data Availability

The data which was analyzed for this study is available from the corresponding author at reasonable request.

## References

[CR1] Agala, C., Fried, B., Thomas, J., Reynolds, H., Lich, K., Whetten, K., Zimmer, C., & Morrissey, J. P. (2019). Reliability, validity and invariance of the simplified medication adherence questionnaire (SMAQ) among HIV-positive women in Ethiopia: A quasi-experimental study. *Bmc Public Health*. 10.21203/RS.2.12427 10.1186/s12889-020-08585-w PMC7189687 32345253

[CR2] Antony, M. M., Cox, B. J., Enns, M. W., Bieling, P. J., & Swinson, R. P. (1998). Psychometric properties of the 42-item and 21-item versions of the Depression anxiety stress scales in clinical groups and a community sample. *Psychological Assessment*, *10*(2), 176–181. 10.1037/1040-3590.10.2.176

[CR3] Belete, H., Mekonen, T., Fekadu, W., Legas, G., & Getnet, A. (2019). Help seeking behavior for problematic substance uses in north-west Ethiopia. *Substance Abuse: Treatment Prevention and Policy*, *14*(1), 1–6. 10.1186/S13011-019-0202-9/TABLES/3 31174556 10.1186/s13011-019-0202-9 PMC6556003

[CR4] Bernstein, D. P., Stein, J. A., Newcomb, M. D., Walker, E., Pogge, D., Ahluvalia, T., Stokes, J., Handelsman, L., Medrano, M., Desmond, D., & Zule, W. (2003). Development and validation of a brief screening version of the Childhood Trauma Questionnaire. *Child Abuse & Neglect*, *27*(2), 169–190. 10.1016/S0145-2134(02)00541-0 12615092 10.1016/s0145-2134(02)00541-0

[CR5] Brezing, C., Ferrara, M., & Freudenreich, O. (2015). The Syndemic illness of HIV and Trauma: Implications for a trauma-informed model of Care. *Psychosomatics*, *56*(2), 107–118. 10.1016/j.psym.2014.10.006 25597836 10.1016/j.psym.2014.10.006

[CR6] Brief, D. J., Bollinger, A. R., Vielhauer, M. J., Berger-Greenstein, J. A., Morgan, E. E., Brady, S. M., Buondonno, L. M., & Keane, T. M. (2010). Understanding the interface of HIV, trauma, post-traumatic stress disorder, and substance use and its implications for health outcomes. *16*(SUPPL. 1). 10.1080/09540120412301315259 10.1080/09540120412301315259 15736824

[CR7] Chandwani, S., Abramowitz, S., Koenig, L. J., Barnes, W., & Angelo, L. D. (2011). *A Multimodal Behavioral Intervention to Impact Adherence and Risk Behavior among Perinatally and Behaviorally HIV-infected Youth: Description, Delivery, and Receptivity of Adolescent Impact*. *23*(3), 222–235. 10.1521/aeap.2011.23.3.222 21696241

[CR8] Closson, K., Dietrich, J. J., Nkala, B., Musuku, A., Cui, Z., Chia, J., Gray, G., Lachowsky, N. J., Hogg, R. S., Miller, C. L., & Kaida, A. (2016). Prevalence, type, and correlates of trauma exposure among adolescent men and women in Soweto, South Africa: Implications for HIV prevention. *Bmc Public Health*, *16*(1), 1–15. 10.1186/s12889-016-3832-0 27884181 10.1186/s12889-016-3832-0 PMC5123224

[CR9] Committee on Child Maltreatment Research, Policy, and Practice for the Next Decade: Phase II; Board on Children, Youth, and F. C. on L. and J. I. of M. N. R. Council (2014). *Consequences of Child Abuse and Neglect - New Directions in Child Abuse and Neglect Research* (A. C. Petersen, J. Joseph, & M. Feit, Eds.). National Academies Press (US). 24757747

[CR10] Cortina, M. A., Sodha, A., Fazel, M., & Ramchandani, P. G. (2012). Prevalence of child mental health problems in Sub-Saharan Africa: A systematic review. In *Archives of Pediatrics and Adolescent Medicine* (Vol. 166, Issue 3, pp. 276–281). 10.1001/archpediatrics.2011.592 10.1001/archpediatrics.2011.592 22393184

[CR11] Daniel, W. W., & Cross, C. L. (2018). *Biostatistics: A foundation for analysis in the health sciences*. Wiley.

[CR12] Dow, D. E., Shayo, A. M., Cunningham, C. K., & Reddy, E. A. (2014). Durability of antiretroviral therapy and predictors of virologic failure among perinatally HIV-infected children in Tanzania: A four-year follow-up. *BMC Infectious Diseases*, *14*(1), 567. 10.1186/s12879-014-0567-3 25373425 10.1186/s12879-014-0567-3 PMC4225040

[CR13] Dow, D. E., Turner, E. L., Shayo, A. M., Mmbaga, B., Cunningham, C. K., & O’Donnell, K. (2016). Evaluating mental health difficulties and associated outcomes among HIV-positive adolescents in Tanzania. *AIDS Care - Psychological and Socio-Medical Aspects of AIDS/HIV*, *28*(7), 825–833. 10.1080/09540121.2016.1139043 10.1080/09540121.2016.1139043 PMC4905805 26837437

[CR14] Dreyer, Z., Henn, C., & Hill, C. (2019). Validation of the Depression anxiety stress Scale-21 (DASS-21) in a non-clinical sample of South African working adults. *Journal of Psychology in Africa*, *29*(4), 346–353. 10.1080/14330237.2019.1647499

[CR15] Enane, L. A., Apondi, E., Omollo, M., Toromo, J. J., Bakari, S., Aluoch, J., Morris, C., Kantor, R., Braitstein, P., Fortenberry, J. D., Nyandiko, W. M., Wools-Kaloustian, K., Elul, B., & Vreeman, R. C. (2021). I just keep quiet about it and act as if everything is alright – the cascade from trauma to disengagement among adolescents living with HIV in western Kenya. *Journal of the International AIDS Society*, *24*(4). 10.1002/JIA2.25695 10.1002/jia2.25695 PMC8035676 33838007

[CR16] Essien, E., Attoe, O., Anake, G., Uwah, E., Aigbomian, E., Eleazu, F., & Udofia, O. (2018). The childhood trauma questionnaire: Validity, reliability and factor structure among adolescents in Calabar, Nigeria. *Nigerian Journal of Medicine*, *27*(3), 252. 10.4103/1115-2613.278787

[CR17] Gamarel, K. E., Nelson, K. M., Brown, L., Fernandez, M. I., & Nichols, S. (2017). The usefulness of the CRAFFT in screening for problematic drug and alcohol use among youth living with HIV. *AIDS and Behavior*, *21*(7), 1868. 10.1007/S10461-016-1640-2 27981399 10.1007/s10461-016-1640-2 PMC5472507

[CR18] Garber, J., & Weersing, V. R. (2010). Comorbidity of anxiety and depression in Youth: Implications for treatment and Prevention. *Clinical Psychology: Science and Practice*, *17*(4), 293–306. 10.1111/j.1468-2850.2010.01221.x 21499544 10.1111/j.1468-2850.2010.01221.x PMC3074295

[CR19] Gerdner, A., & Allgulander, C. (2009). Psychometric properties of the Swedish version of the Childhood Trauma Questionnaire - Short Form (CTQ-SF). *Nordic Journal of Psychiatry*, *63*(2), 160–170. 10.1080/08039480802514366 19021077 10.1080/08039480802514366

[CR20] Heim, C., Newport, D. J., Mletzko, T., Miller, A. H., & Nemeroff, C. B. (2008). The link between childhood trauma and depression: Insights from HPA axis studies in humans. *Psychoneuroendocrinology*, *33*(6), 693–710. 10.1016/j.psyneuen.2008.03.008 18602762 10.1016/j.psyneuen.2008.03.008

[CR21] Hirasen, K., Evans, D., Jinga, N., Grabe, R., Turner, J., Mashamaite, S., Long, L. C., & Fox, M. P. (2020). Using a self-administered electronic adherence questionnaire to identify poor adherence amongst adolescents and young adults on first-line antiretroviral therapy in Johannesburg, South Africa. *Patient Preference and Adherence*, *14*, 133–151. 10.2147/PPA.S210404 32021124 10.2147/PPA.S210404 PMC6987979

[CR22] Humphreys, K. L., LeMoult, J., Wear, J. G., Piersiak, H. A., Lee, A., & Gotlib, I. H. (2020). Child maltreatment and depression: A meta-analysis of studies using the Childhood Trauma Questionnaire. *Child Abuse and Neglect*, *102*. 10.1016/j.chiabu.2020.104361 10.1016/j.chiabu.2020.104361 PMC7081433 32062423

[CR23] Iacob, S. A., Iacob, D. G., & Jugulete, G. (2017). Improving the adherence to antiretroviral therapy, a difficult but essential task for a successful HIV treatment-clinical points of view and practical considerations. In *Frontiers in Pharmacology* (Vol. 8, Issue NOV). Frontiers Media S.A. 10.3389/fphar.2017.00831 10.3389/fphar.2017.00831 PMC5703840 29218008

[CR24] Infurna, M. R., Reichl, C., Parzer, P., Schimmenti, A., Bifulco, A., & Kaess, M. (2016). Associations between depression and specific childhood experiences of abuse and neglect: A meta-analysis. *Journal of Affective Disorders*, *190*, 47–55. 10.1016/J.JAD.2015.09.006 26480211 10.1016/j.jad.2015.09.006

[CR25] Jacobsen, L. K., Southwick, S. M., & Kosten, T. R. (2001). Substance use disorders in patients with posttraumatic stress disorder: A review of the literature. *American Journal of Psychiatry*, *158*(8), 1184–1190. 10.1176/APPI.AJP.158.8.1184/ASSET/IMAGES/LARGE/J52F1.JPEG 11481147 10.1176/appi.ajp.158.8.1184

[CR26] Jewkes, R. K., Dunkle, K., Nduna, M., Jama, P. N., & Puren, A. (2010). Associations between childhood adversity and depression, substance abuse and HIV and HSV2 incident infections in rural South African youth. *Child Abuse and Neglect*, *34*(11), 833–841. 10.1016/j.chiabu.2010.05.002 20943270 10.1016/j.chiabu.2010.05.002 PMC2981623

[CR27] Julnes, P. S., Auh, S., Krakora, R., Withers, K., Nora, D., Matthews, L., Steinbach, S., Snow, J., Smith, B., Nath, A., Morse, C., & Kapetanovic, S. (2016). The Association Between Post-traumatic Stress Disorder and Markers of Inflammation and Immune Activation in HIV-Infected Individuals With Controlled Viremia From Section of Infections of the Nervous System. In *Psychosomatics* (Vol. 57). www.psychosomaticsjournal.org. 10.1016/j.psym.2016.02.015 PMC4902734 27095586

[CR28] Kahana, S. Y., Rohan, J., Allison, S., Frazier, T. W., & Drotar, D. (2013). A meta-analysis of adherence to antiretroviral therapy and virologic responses in HIV-infected children, adolescents, and young adults. *AIDS and Behavior*, *17*(1), 41–60. 10.1007/s10461-012-0159-4 22411426 10.1007/s10461-012-0159-4

[CR29] Kamau, J. W., Kuria, W., Mathai, M., Atwoli, L., & Kangethe, R. (2012). Psychiatric morbidity among HIV-infected children and adolescents in a resource-poor Kenyan urban community. *Aids Care*, *24*(7), 836–842. 10.1080/09540121.2011.644234 22292795 10.1080/09540121.2011.644234

[CR30] Kim, D., Bae, H., Han, C., Oh, H. Y., & MacDonald, K. (2013). Psychometric properties of the Childhood Trauma Questionnaire-Short Form (CTQ-SF) in Korean patients with schizophrenia. *Schizophrenia Research*, *144*(1–3), 93–98. 10.1016/j.schres.2012.12.020 23352775 10.1016/j.schres.2012.12.020

[CR31] Kim, S. H., Gerver, S. M., Fidler, S., & Ward, H. (2014). Adherence to antiretroviral therapy in adolescents living with HIV: Systematic review and meta-analysis. *Aids (London, England)*, *28*(13), 1945–1956. 10.1097/QAD.0000000000000316 24845154 10.1097/QAD.0000000000000316 PMC4162330

[CR32] Kimerling, R., Calhoun, K. S., Forehand, R., Armistead, L., Morse, E., Morse, P., Clark, R., & Clark, L. (1999). Traumatic stress in HIV-infected women. *AIDS Education and PreventionTraum*, *11*(4), 321–330. 10494356

[CR35] Knight, J. R., Shrier, L. A., Bravender, T. D., Farrell, M., Bilt, J., Vander, & Shaffer, H. J. (1999). A new brief screen for adolescent substance abuse. *Archives of Pediatrics and Adolescent Medicine*, *153*(6), 591–596. 10.1001/archpedi.153.6.591 10357299 10.1001/archpedi.153.6.591

[CR34] Knight, J. R., Sherritt, L., Shrier, L. A., Harris, S. K., & Chang, G. (2002). Validity of the CRAFFT substance abuse screening test among adolescent clinic patients. *Archives of Pediatrics and Adolescent Medicine*, *156*(6), 607–614. 10.1001/archpedi.156.6.607 12038895 10.1001/archpedi.156.6.607

[CR33] Knight, J. R., Sherritt, L., Harris, S. K., Gates, E. C., & Chang, G. (2003). Validity of brief alcohol screening tests among adolescents: A comparison of the AUDIT, POSIT, CAGE, and CRAFFT. *Alcoholism: Clinical and Experimental Research*, *27*(1), 67–73. 10.1111/j.1530-0277.2003.tb02723.x 12544008 10.1097/01.ALC.0000046598.59317.3A

[CR36] Knobel, H., Alonso, J., Casado, J. L., Collazos, J., González, J., Ruiz, I., Kindelan, J. M., Carmona, A., Juega, J., & Ocampo, A. (2002). Validation of a simplified medication adherence questionnaire in a large cohort of HIV-infected patients: The GEEMA study. *Aids*, *16*(4), 605–613. 10.1097/00002030-200203080-00012 11873004 10.1097/00002030-200203080-00012

[CR37] Lansford, J. E., Godwin, J., Tirado, L. M. U., Zelli, A., Al-Hassan, S. M., Bacchini, D., Bombi, A. S., Bornstein, M. H., Chang, L., Deater-Deckard, K., Di Giunta, L., Dodge, K. A., Malone, P. S., Oburu, P., Pastorelli, C., Skinner, A. T., Sorbring, E., Tapanya, S., & Alampay, L. P. (2015). Individual, Family, and culture level contributions to child physical abuse and neglect: A longitudinal study in nine countries. *Development and Psychopathology*, *27*(4 Pt 2), 1417. 10.1017/S095457941500084X 26535934 10.1017/S095457941500084X PMC4839471

[CR38] Le, M. T. H., Tran, T. D., Holton, S., Nguyen, H. T., Wolfe, R., & Fisher, J. (2017). Reliability, convergent validity and factor structure of the DASS-21 in a sample of Vietnamese adolescents. *Plos One*, *12*(7). 10.1371/journal.pone.0180557 10.1371/journal.pone.0180557 PMC5516980 28723909

[CR39] LeGrand, S., Reif, S., Sullivan, K., Murray, K., Barlow, M. L., & Whetten, K. (2015). A review of recent literature on Trauma among individuals living with HIV. In *current HIV/AIDS reports*. *Current Medicine Group LLC 1*, *12*(4), 397–405. 10.1007/s11904-015-0288-2 10.1007/s11904-015-0288-2 PMC4837695 26419376

[CR40] Leserman, J. (2008). Role of depression, stress, and trauma in HIV disease progression. *Psychosomatic Medicine*, *70*(5), 539–545. 10.1097/PSY.0b013e3181777a5f 18519880 10.1097/PSY.0b013e3181777a5f

[CR41] Lovibond, S. H., & Lovibond, P. F. (1995). *Manual for the Depression anxiety stress scales* (2nd ed.). Psychology Foundation.

[CR43] Lowenthal, E., Lawler, K., Harari, N., Moamogwe, L., Masunge, J., Masedi, M., Matome, B., Seloilwe, E., & Gross, R. (2012). Rapid psychosocial function screening test identified treatment failure in HIV + African youth. *AIDS Care - Psychological and Socio-Medical Aspects of AIDS/HIV*, *24*(6), 722–727. 10.1080/09540121.2011.644233 10.1080/09540121.2011.644233 PMC3345310 22292411

[CR42] Lowenthal, E., Bakeera-Kitaka, S., Marukutira, T., Chapman, J., Goldrath, K., & Ferrand, R. A. (2014). Perinatally acquired HIV infection in adolescents from sub-Saharan Africa: A review of emerging challenges. In *The Lancet Infectious Diseases* (Vol. 14, Issue 7, pp. 627–639). Lancet Publishing Group. 10.1016/S1473-3099(13)70363-3 10.1016/S1473-3099(13)70363-3 PMC4074242 24406145

[CR44] Mabaso, M., Maseko, G., Sewpaul, R., Naidoo, I., Jooste, S., Takatshana, S., Reddy, T., Zuma, K., & Zungu, N. (2021). Trends and correlates of HIV prevalence among adolescents in South Africa: Evidence from the 2008, 2012 and 2017 South African National HIV Prevalence, incidence and Behaviour surveys. *AIDS Research and Therapy*, *18*(1), 1–8. 10.1186/S12981-021-00422-3/TABLES/4 34906170 10.1186/s12981-021-00422-3 PMC8670218

[CR45] McLaughlin, K. A., Green, J. G., Gruber, M. J., Sampson, N. A., Zaslavsky, A. M., & Kessler, R. C. (2012). Childhood adversities and first onset of psychiatric disorders in a national sample of US adolescents. *Archives of General Psychiatry*, *69*(11), 1151–1160. 10.1001/archgenpsychiatry.2011.2277 23117636 10.1001/archgenpsychiatry.2011.2277 PMC3490224

[CR46] Mellins, C. A., & Malee, K. M. (2013). Understanding the mental health of youth living with perinatal HIV infection: Lessons learned and current challenges. *Journal of the International AIDS Society*, *16*(1), 18593. 10.7448/IAS.16.1.18593 23782478 10.7448/IAS.16.1.18593 PMC3687078

[CR47] Moya, E., Larson, L. M., Stewart, R. C., Fisher, J., Mwangi, M. N., & Phiri, K. S. (2022). Reliability and validity of depression anxiety stress scale (DASS)-21 in screening for common mental disorders among postpartum women in Malawi. *Bmc Psychiatry*, *22*(1). 10.1186/s12888-022-03994-0 10.1186/s12888-022-03994-0 PMC9128196 35606733

[CR48] Mullan, L. E., Mullan, P. C., & Anabwani, G. M. (2015a). Psychosocial issues among children and adolescents in an integrated paediatric HIV psychology service in Botswana. *Journal of Psychology in Africa*, *25*(2), 160–163. 10.1080/14330237.2015.1021534

[CR49] Mullan, L. E., Mullan, P. C., & Anabwani, G. M. (2015b). Psychosocial issues among children and adolescents in an integrated paediatric HIV psychology service in Botswana. *Journal of Psychology in Africa*, *25*(2), 160–163. 10.1080/14330237.2015.1021534

[CR50] Nachega, J. B., Hislop, M., Nguyen, H., Dowdy, D. W., Chaisson, R. E., Regensberg, L., Cotton, M., & Maartens, G. (2009). Antiretroviral therapy adherence, virologic and immunologic outcomes in adolescents compared with adults in Southern Africa. *Journal of Acquired Immune Deficiency Syndromes*, *51*(1), 65–71. 10.1097/QAI.0b013e318199072e 19282780 10.1097/QAI.0b013e318199072e PMC2674125

[CR51] Nightingale, V. R., Sher, T. G., Mattson, M., Thilges, S., & Hansen, N. B. (2011). The effects of traumatic stressors and HIV-related trauma symptoms on health and health related quality of life. *AIDS and Behavior*, *15*(8), 1870–1878. 10.1007/S10461-011-9980-4/FIGURES/1 21667297 10.1007/s10461-011-9980-4 PMC3629911

[CR52] Oladeji, B. D., Makanjuola, V. A., & Gureje, O. (2010). Family-related adverse childhood experiences as risk factors for psychiatric disorders in Nigeria. *British Journal of Psychiatry*, *196*(3), 186–191. 10.1192/bjp.bp.109.063677 10.1192/bjp.bp.109.063677 PMC2830054 20194539

[CR53] Olashore, A. A., Paruk, S., Tshume, O., & Chiliza, B. (2022). Depression and suicidal behavior among adolescents living with HIV in Botswana: A cross-sectional study. *Child and Adolescent Psychiatry and Mental Health*, *16*(1), 1–9. 10.1186/s13034-022-00492-9 35906651 10.1186/s13034-022-00492-9 PMC9336130

[CR54] Pence, B. W., Shirey, K., Whetten, K., Agala, B., Itemba, D., Adams, J., Whetten, R., Yao, J., & Shao, J. (2012). Prevalence of psychological trauma and association with current health and functioning in a sample of HIV-infected and HIV-uninfected Tanzanian adults. *Plos One*, *7*(5), e36304. 10.1371/journal.pone.0036304 22606252 10.1371/journal.pone.0036304 PMC3351441

[CR55] Ramaiya, M. K., Sullivan, K. A., O’ Donnell, K., Cunningham, C. K., Shayo, A. M., Mmbaga, B. T., & Dow, D. E. (2016). A qualitative exploration of the Mental Health and Psychosocial contexts of HIV-Positive adolescents in Tanzania. *PLOS ONE*, *11*(11), e0165936. 10.1371/journal.pone.0165936 27851797 10.1371/journal.pone.0165936 PMC5112865

[CR56] Remien, R. H., Stirratt, M. J., Nguyen, N., Robbins, R. N., Pala, A. N., & Mellins, C. A. (2019). Mental health and HIV/AIDS: The need for an integrated response. *AIDS* (Vol. 33, pp. 1411–1420). Lippincott Williams and Wilkins. 910.1097/QAD.0000000000002227 10.1097/QAD.0000000000002227 PMC6635049 30950883

[CR57] Scott, K. M., Von Korff, M., Angermeyer, M. C., Benjet, C., Bruffaerts, R., De Girolamo, G., Haro, J. M., Lépine, J. P., Ormel, J., Posada-Villa, J., Tachimori, H., & Kessler, R. C. (2011). Association of childhood adversities and early-onset mental disorders with adult-onset chronic physical conditions. *Archives of General Psychiatry*, *68*(8), 838–844. 10.1001/archgenpsychiatry.2011.77 21810647 10.1001/archgenpsychiatry.2011.77 PMC3402030

[CR58] Seloilwe, E. S., & Thupayagale-Tshweneagae, G. (2009). Sexual abuse and violence among adolescent girls in Botswana: A mental health perspective. *Issues in Mental Health Nursing*, *30*(7), 456–459. 10.1080/01612840903039367 19544130 10.1080/01612840903039367

[CR59] Spies, G., Kidd, M., & Seedat, S. (2019). A factor analytic study of the Childhood Trauma Questionnaire-Short Form in an all-female South African sample with and without HIV infection. *Child Abuse & Neglect*, *92*, 157. 10.1016/J.CHIABU.2019.04.002 30981158 10.1016/j.chiabu.2019.04.002 PMC8853848

[CR60] Szabó, M. (2010). The short version of the Depression anxiety stress scales (DASS-21): Factor structure in a young adolescent sample. *Journal of Adolescence*, *33*(1), 1–8. 10.1016/j.adolescence.2009.05.014 19560196 10.1016/j.adolescence.2009.05.014

[CR61] Tirfeneh, E., & Srahbzu, M. (2020). Depression and Its Association with Parental Neglect among Adolescents at Governmental High Schools of Aksum Town, Tigray, Ethiopia, 2019: A Cross Sectional Study. *Depression Research and Treatment*, *2020*. 10.1155/2020/6841390 10.1155/2020/6841390 PMC7210544 32411458

[CR62] UNAIDS (2016). *AIDSInfo data*. http://aidsinfo.unaids.org/

[CR63] UNAIDS (2017). Ending Aids: Progress Towards the 90-90-90 Targets. In *Global Aids Update*. https://doi.org/UNAIDS/JC2900E

[CR64] UNAIDS (2018). *AIDSInfo 2018 data*.

[CR66] UNICEF (2019b). *HIV | UNICEF Botswana*. https://www.unicef.org/botswana/hiv

[CR65] UNICEF (2019a). *Children, HIV and AIDS Regional snapshot : Sub-Saharan Africa*. https://data.unicef.org/topic/hiv-aids

[CR67] Uthman, O. A., Magidson, J. F., Safren, S. A., & Nachega, J. B. (2014). Depression and adherence to antiretroviral therapy in low-, middle- and high-income countries: A systematic review and meta-analysis. *Current HIV/AIDS Reports*, *11*(3), 291–307. 10.1007/s11904-014-0220-1 25038748 10.1007/s11904-014-0220-1 PMC4359613

[CR68] Vreeman, R. C., McCoy, B. M., & Lee, S. (2017). Mental health challenges among adolescents living with HIV. *Journal of the International AIDS Society*, *20*(3), 100–109. 10.7448/IAS.20.4.21497 10.7448/IAS.20.4.21497 PMC5577712 28530045

[CR69] Waldron, E. M., Burnett-Zeigler, I., Wee, V., Ng, Y. W., Koenig, L. J., Pederson, A. B., Tomaszewski, E., & Miller, E. S. (2021). Mental Health in Women Living with HIV: The unique and unmet needs. *Journal of the International Association of Providers of AIDS Care (Vol*, *20*, 1–18. 10.1177/2325958220985665 10.1177/2325958220985665 PMC7829520 33472517

[CR70] Wang, T., Fu, H., Kaminga, A. C., Li, Z., Guo, G., Chen, L., & Li, Q. (2018). Prevalence of depression or depressive symptoms among people living with HIV/AIDS in China: A systematic review and meta-analysis. *Bmc Psychiatry*, *18*(1), 1–14. 10.1186/s12888-018-1741-8 29855289 10.1186/s12888-018-1741-8 PMC5984474

[CR71] Whetten, K., Reif, S., Whetten, R., & Murphy-Mcmillan, L. K. (2008). Trauma, mental health, distrust, and stigma among HIV-positive persons: Implications for effective care. *Psychosomatic Medicine*, *70*(5), 531–538. 10.1097/PSY.0b013e31817749dc 18541904 10.1097/PSY.0b013e31817749dc

[CR72] World Health Organisation (2020). *Adolescent health*. https://www.who.int/southeastasia/health-topics/adolescent-health

[CR73] Yang, E., Mphele, S., Moshashane, N., Bula, B., Chapman, J., Okatch, H., Pettitt, E., Tshume, O., Marukutira, T., Anabwani, G., & Lowenthal, E. (2018). Distinctive barriers to antiretroviral therapy adherence among non-adherent adolescents living with HIV in Botswana. *Aids Care*, *30*(2), 224–231. 10.1080/09540121.2017.1344767.Distinctive 28643572 10.1080/09540121.2017.1344767 PMC6083824

