# Supplementary material for: Association between Childhood Trauma, Mental Health Symptoms and Adherence Among Youth Living with HIV in Botswana
Source: J Child Adolesc Trauma. 2024 Aug 29;17(4):1079–89. doi: 10.1007/s40653-024-00658-x (PMC11646251; doi:10.1007/s40653-024-00658-x)
Supplement: Supplementary file 1 — Supplementary Material 1 [file 40653_2024_658_MOESM1_ESM.docx]

| Table 1 : Bivariate regression analysis of mental health symptoms, substance use, non-adherence, and childhood trauma exposure | | | | | |
| --- | --- | --- | --- | --- | --- |
|  | Depression |  |  |  |  |
|  | OR | SE | p | 95% CI | |
| ≥1 Childhood trauma exposure | 3.64 | 1.49 | 0.002* | 1.63 | 8.12 |
| Emotional abuse | 3.81 | 2.51 | 0.043* | 1.04 | 13.89 |
| Physical abuse | 6.78 | 4.22 | 0.002* | 2.00 | 22.99 |
| Sexual abuse | 2.46 | 1.31 | 0.090 | 0.87 | 6.96 |
| Emotional neglect | 1.95 | 0.86 | 0.131 | 0.82 | 4.64 |
| Physical neglect | 2.67 | 1.07 | 0.014* | 1.22 | 5.87 |
|  |  |  |  |  |  |
|  | Anxiety |  |  |  |  |
|  | OR | SE | p | 95% CI | |
| ≥1 Childhood trauma exposure | 2.51 | 0.96 | 0.016* | 1.19 | 5.32 |
| Emotional abuse | 4.36 | 3.07 | 0.037* | 1.09 | 17.37 |
| Physical abuse | 7.24 | 4.91 | 0.003* | 1.92 | 27.31 |
| Sexual abuse | 1.32 | 0.70 | 0.595 | 0.47 | 3.71 |
| Emotional neglect | 2.34 | 1.03 | 0.053* | 0.99 | 5.55 |
| Physical neglect | 1.90 | 0.74 | 0.098 | 0.89 | 4.07 |
|  |  |  |  |  |  |
|  | Stress |  |  |  |  |
|  | OR | SE | p | 95% CI | |
| ≥1 Childhood trauma exposure | 2.49 | 1.34 | 0.090 | 0.87 | 7.15 |
| Emotional abuse | 3.84 | 2.64 | 0.051* | 0.99 | 14.81 |
| Physical abuse | 2.34 | 1.52 | 0.192 | 0.65 | 8.37 |
| Sexual abuse | 2.85 | 1.74 | 0.085 | 0.86 | 9.42 |
| Emotional neglect | 1.80 | 1.00 | 0.292 | 0.60 | 5.33 |
| Physical neglect | 1.51 | 0.78 | 0.428 | 0.55 | 4.17 |
|  |  |  |  |  |  |
|  | Problematic substance use | | |  |  |
|  | OR | SE | p | 95% CI | |
| ≥1 Childhood trauma exposure | 3.65 | 1.71 | 0.006* | 1.45 | 9.16 |
| Emotional abuse | 2.00 | 1.34 | 0.300 | 0.54 | 7.41 |
| Physical abuse | 1.76 | 1.05 | 0.343 | 0.55 | 5.67 |
| Sexual abuse | 4.91 | 2.70 | 0.004* | 1.68 | 14.40 |
| Emotional neglect | 1.42 | 0.69 | 0.473 | 0.54 | 3.70 |
| Physical neglect | 2.14 | 0.94 | 0.084 | 0.90 | 5.06 |
|  |  |  |  |  |  |
|  | Non-adherence | |  |  |  |
|  | OR | SE | p | 95% CI | |
| ≥1 Childhood trauma exposure | 3.51 | 1.36 | 0.001* | 1.65 | 7.50 |
| Emotional abuse | 5.53 | 4.46 | 0.034* | 1.14 | 26.82 |
| Physical abuse | 3.41 | 2.10 | 0.047* | 1.02 | 11.41 |
| Sexual abuse | 5.71 | 3.83 | 0.009* | 1.53 | 21.28 |
| Emotional neglect | 2.89 | 1.32 | 0.020* | 1.18 | 7.08 |
| Physical neglect | 2.53 | 1.00 | 0.018* | 1.17 | 5.48 |

*significant association (p < 0.05)
